# Intradiaphragmatic pulmonary sequestrations: a surgical challenge. Case series

**DOI:** 10.3389/fsurg.2023.1181007

**Published:** 2023-05-25

**Authors:** Chiara Oreglio, Francesca Tocchioni, Marco Ghionzoli, Annamaria Buccoliero, Antonino Morabito, Francesco Morini

**Affiliations:** ^1^ Department of Neuroscience, Psychology, Drug Research and Child Health (NEUROFARBA), University of Florence, Florence, Italy; ^2^ Department of Pediatric and Neonatal Surgery, Meyer Children’s Hospital IRCCS, Florence, Italy; ^3^ Department of Pediatric and Adolescent Surgery, University of Pisa, Pisa, Italy; ^4^ Department of Surgical, Medical, Molecular Pathology and of the Critic Area, University of Pisa, Pisa, Italy; ^5^ Department of Pathology, Meyer Children’s Hospital IRCCS, Florence, Italy

**Keywords:** children, congenital lung malformation, CPAM, bronchopulmonary sequestration, thoracoscopy, laparoscopy

## Abstract

Bronchopulmonary sequestrations (BPSs) are rare congenital anomalies characterized by non-functioning embryonic lung tissue receiving anomalous blood supply. They are most commonly located within the thorax (supradiaphragmatic) or into the abdominal cavity (infradiaphragmatic). Intradiaphragmatic extralobar BPs (IDEPS) are an exceptionally rare finding, representing a diagnostic and operative challenge. We report three cases of IDEPS and their surgical management, describing our experience and approach to such rare clinical entities. From 2016 to 2022, we treated 3 cases of IDEPS. Surgical techniques, histopathological findings and clinical outcomes were retrospectively evaluated for each case and compared. Three different surgical techniques were used to approach each lesion, from open thoracotomy to a combined laparoscopic and thoracoscopic approach. Histopathological analysis of the specimens revealed hybrid pathological features, proper of both congenital pulmonary airway malformation (CPAM) and extralobar pulmonary sequestration. IDEPS represent a surgical challenge for pediatric surgeons, given their complex surgical planning. In our experience, the thoracoscopic approach is safe and feasible when performed by trained surgeons, even though a combined thoracoscopic-laparoscopic approach allows for optimal vessels control. The presence of CPAM elements within the lesions supports their surgical removal. Additional studies are required to better characterize IDEPS and their management.

## 1. Introduction

Bronchopulmonary sequestration (BPS) is a rare congenital lung malformation (CLM) characterized by non-functioning embryonic lung tissue deriving its blood supply from one or more anomalous systemic arteries (1). Based on their location relative to the diaphragm, BPS can be classified as supra-diaphragmatic (intralobar or extralobar), infradiaphragmatic, or, more rarely, intradiaphragmatic. Intralobar sequestration (ILS) is covered by normal lungs' visceral pleura, while extralobar sequestration (ELS) is not and can be found within the thoracic cavity (intrathoracic) or outside (ectopic) (2). Intradiaphragmatic extralobar BPS (IDEPS) are an exceedingly rare finding and very few cases have been described in the literature (3–5). These peculiar lesions are challenging from a surgical point of view given the unusual location and the consequent complexity in choosing the best approach to reach optimal surgical outcome. Not only identification of the lesion itself may be arduous, being hidden within the diaphragm muscle. Difficulty also resides in safely identifying and sectioning the feeding vessel(s), to avoid severe vascular complications.

No clear indication exists on whether IDEPS should be operated upon and their optimal approach. We hereby report our center's experience with the management of patients with IDEPS to describe the surgical techniques used and their histopathological peculiarities.

## 2. Case presentation

Between 2016 and 2022 we managed 36 consecutive patients with CLM. Twelve had congenital pulmonary airway malformations, 18 had BPS (13 hybrid lesions), 3 had congenital lobar emphysema, 2 a bronchogenic cyst, and one a necrotic single pulmonary cyst. Out of 18 patients with BPS, 13 were ILS (10 hybrid lesions) and 5 were ELS, of which 3 IDEPS.

The details of patients with IDEPS are as follows:

### 2.1. Case 1

**Figure 1:**
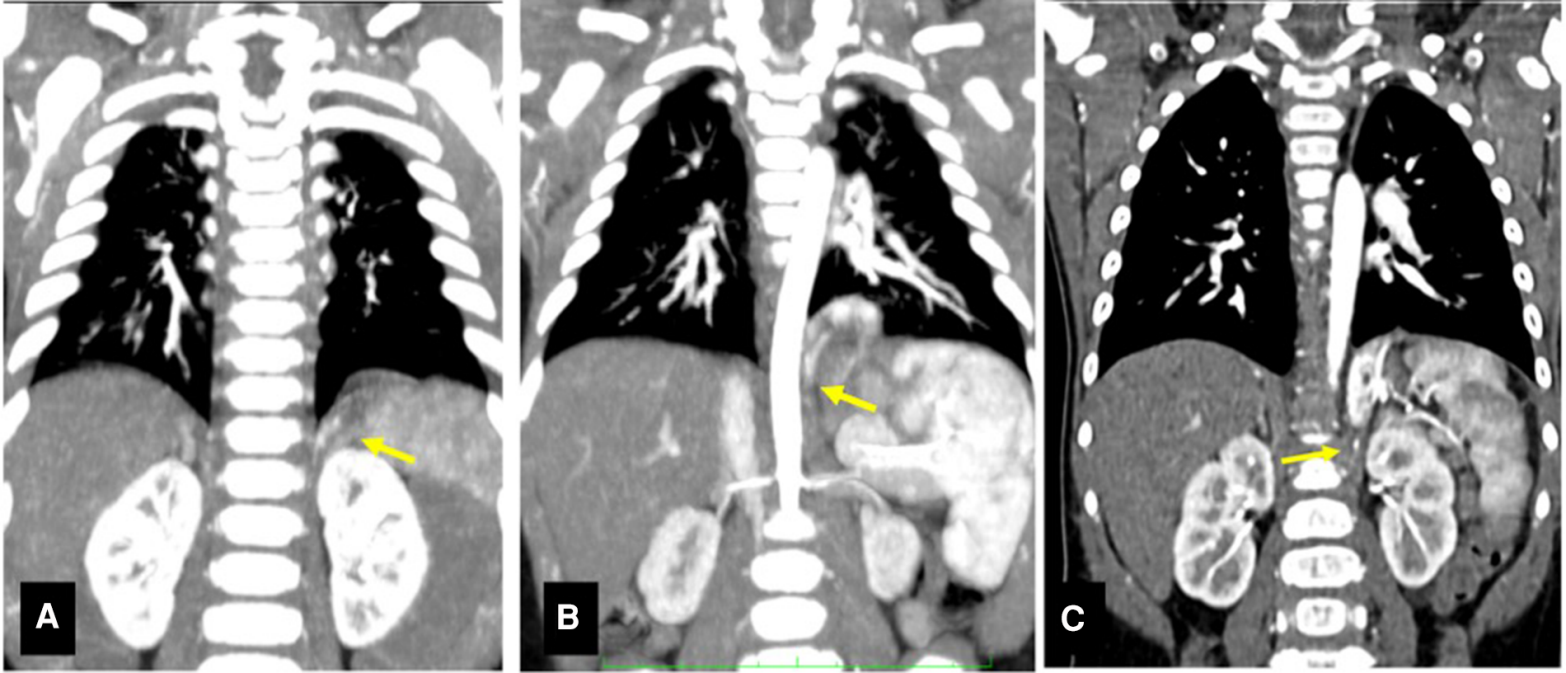
CT scans of presented cases. (**A**) CT scan Case 1. Structural alteration in the left lower lobe with increased parenchyma opacity, no signs of ventilation (1.5 cm × 2.7 cm × 1.5 cm), extending within the diaphragm. Arrow: small diaphragmatic arterial vessel originating from the celiac trunk is visible. (**B**) CT scan Case 2. Solid formation in the left costo-vertebral diaphragmatic angle (2.0 cm × 2.0 cm × 1.7 cm), regular margins and no clear connection with the bronchial tree. Arrow: artery originating from the celiac trunk (4.5 mm below its aortic origin). (**C**) CT scan Case 3. Solid intradiafragmatic mass with regular margins (3.3 cm × 1.5 cm × 2.9 cm). Arrow: systemic arterial supply with arteries from the celiac trunk.

A 5-month-old, asymptomatic, infant was referred for surgical evaluation of a left lower thoracic lesion detected on prenatal ultrasound (US). An angio-computed tomography (CT) scan was performed at birth, which confirmed the presence of the lesion located within the diaphragm and showed incidental finding of emphysema of the left lower lobe (Figure 1A). An aberrant artery originating from the celiac trunk was identified supplying the mass within the diaphragm. Parents were instructed regarding the conservative approach for the emphysematous lesion (6) and the possibility to choose for removal or close follow up of the intradiaphragmatic mass. After surgical counselling, parents' preference was to remove the lesion, together with the emphysematous lobe. Therefore, removal of the mass together with left lower lobectomy was performed at 6 months of life.

A thoracotomy on the fourth intercostal space was performed. Following formal left lower lobectomy, dissection of the diaphragmatic muscular fibers was performed: the BPS was pulled up, allowing the identification and sectioning of its large feeding aberrant artery, together with concomitant minor vessels. After the BPS was removed, the diaphragm was sutured with single-line non-absorbable stitches. A chest tube was left and removed on postoperative day (POD). The postoperative course was uneventful, and the patient was discharged at home on the 7th POD. At 2 years' follow-up, the patient was thriving well, free of symptoms and chest x-ray was normal.

### 2.2. Case 2

A 7-month-old asymptomatic infant was referred after prenatal diagnosis of left lung malformation, not otherwise specified. Pregnancy and delivery were regular, and the patient was asymptomatic at birth. Apgar score was 9 and 9 at 1 and 5 min, respectively. The neonatal period was uneventful too. A thoracic CT scan was performed which described a left lower lesion, fed by an aberrant artery arising from the celiac trunk (Figure 1B).

For the query diagnosis of left ELS, a thoracoscopic resection was planned at 7 months of age. However, at thoracoscopy, the postero-lateral portion of the lesion could be reached only partially, and a bulging of the diaphragm was identified. Therefore, the operation was converted to thoracotomy on the fourth intercostal space. This allowed better visualization of the diaphragmatic bulging in its postero-lateral portion that was covered by diaphragmatic serosa. The mass was isolated, the feeding artery arising from the celiac trunk was identified and clipped, and the BPS was resected. The diaphragmatic breach was sutured through single-line non-absorbable stitches. The postoperative course was uneventful. The patient was discharged home on POD 10. At 1-year follow-up, the patient was free of symptoms.

### 2.3. Case 3

A 7-month-old asymptomatic infant was referred to our Surgical Department with a prenatal diagnosis of CLM. A prenatal magnetic resonance imaging (MRI) study performed at 30th gestational week showed an intradiaphragmatic mass, with clear margins, imprinting the lower lobe of the left lung and apparently extending below the diaphragm, medial and posterior to the stomach. Pregnancy and vaginal delivery were uneventful. Again, the patient was asymptomatic at birth. Apgar score was 9- and 9 at 1 and 5 min, respectively. The neonatal period was unremarkable. A post-natal CT scan with contrast medium performed at 12 months of age confirmed the lesion, apparently limited to the left diaphragmatic pillar and receiving systemic blood supply from the celiac trunk, the lumbar arteries, the diaphragmatic arteries and a single small vessel from the splenic artery, thereby suggesting IDEPS (Figure 1C). Surgical removal was therefore planned at 1 year of age.

Given the systemic arterial supply originating mainly within the abdomen, the decision was made for an initial laparoscopic approach with readily available conversion into thoracoscopy, if necessary.

**Figure 2:**
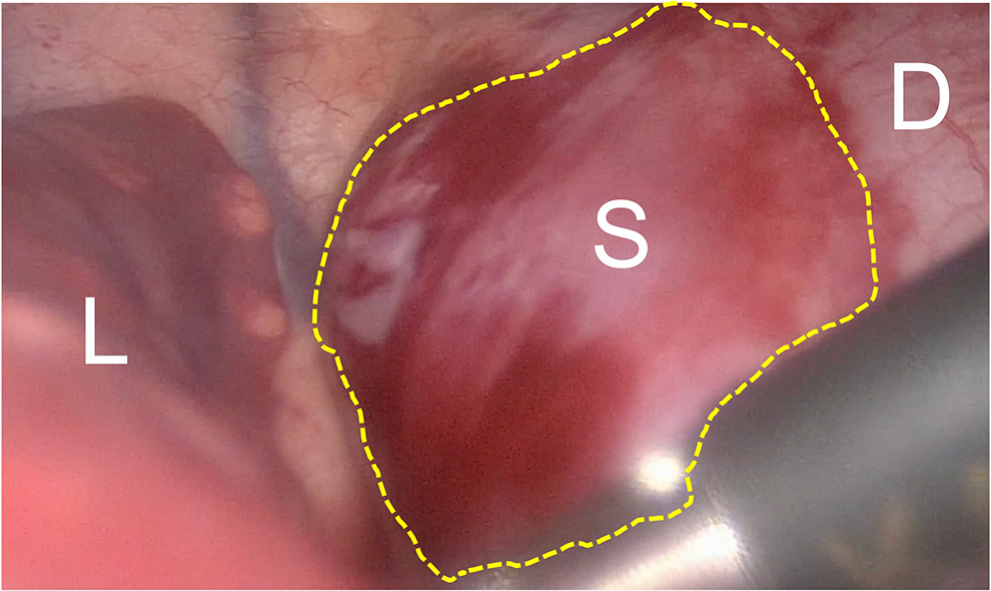
Toracoscopic view of IDEPS. Toracoscopic view of the IDEPS, with bulging of the diaphragm. L, lung; S, sequestration; D, diaphragm.

During laparoscopy, two small arteries and one vein penetrating the diaphragm were identified posterior to the stomach and medial to the spleen. They were clipped and sectioned. However, even after careful dissection of diaphragmatic muscle, the mass was not clearly identified. Therefore, a thoracoscopy was performed, highlighting a bulging of the diaphragm in the left paravertebral region (Figure 2). The diaphragmatic pleura was incised above the prominence, allowing the clear identification of the mass and its further isolation. Three additional arterial vessels were identified, one of which was the main feeding vessel originating from the celiac trunk. They were clipped and sectioned. Reconstruction of the diaphragm was performed with three non-absorbable sutures using Roeder's knots. No intraoperative complications were reported. The postoperative course was uneventful, and the patient was discharged at home on the 5th POD. At 4-months follow-up, the patient was asymptomatic.

**Figure 3:**
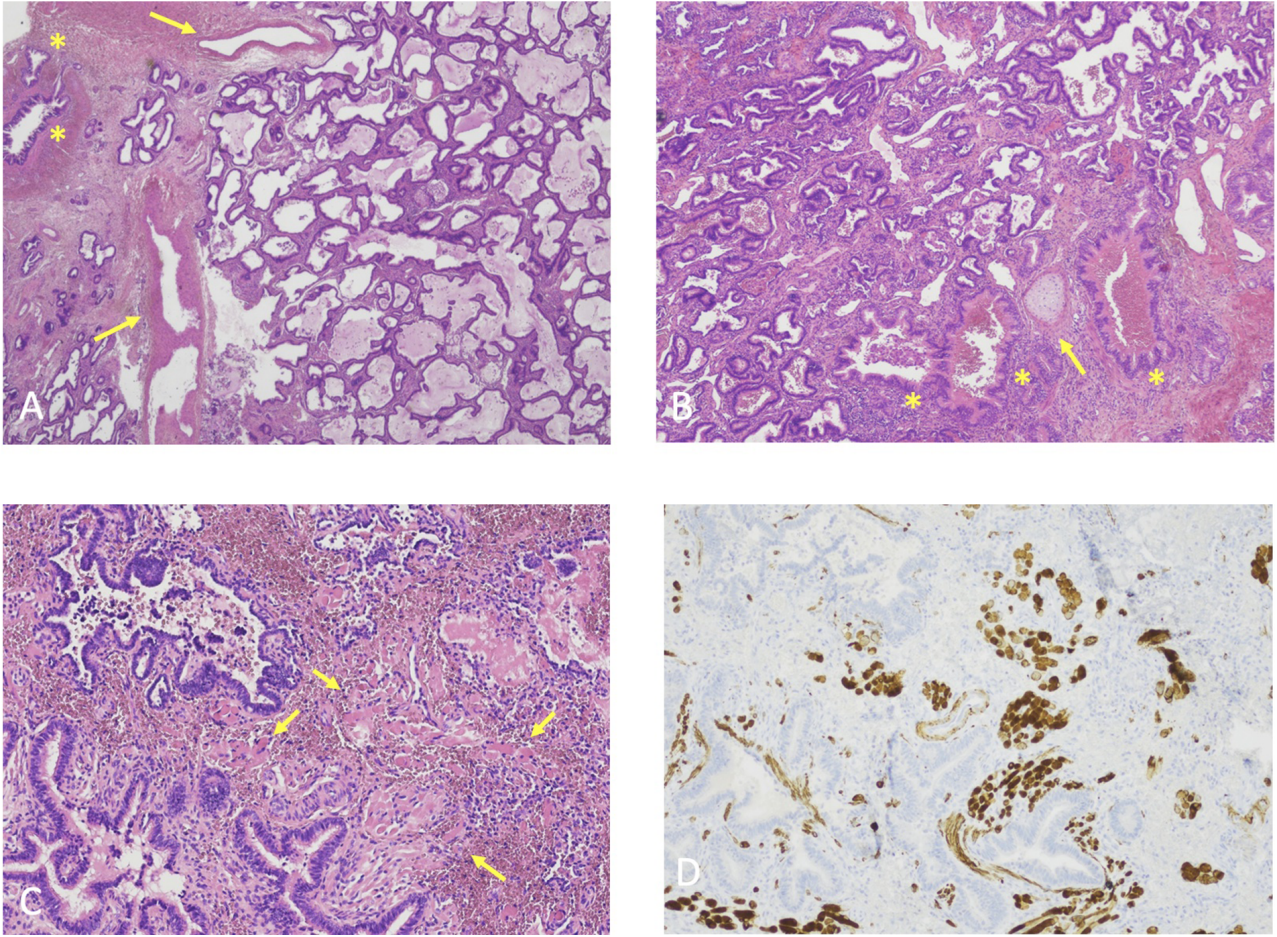
(**A**) Histological features case 1, hematoxylin-eosin, original magnification 2X. Back-to-back cystic spaces lined by pseudostratified ciliated columnar epithelium consistent with CPAM. Asterisks: bronchioles; Arrows: aberrant vessels. (**B**) Histological features Case 2, Hematoxylin-eosin, original magnification 4X. Back-to-back cystic spaces lined by pseudostratified ciliated columnar epithelium consistent with CPAM. Asterisks: bronchioles; Arrows: cartilage plate. (**C**) Histological features Case 3, Hematoxylin-eosin, original magnification 10X. Irregularly shaped airways cystic spaces lined by pseudostratified ciliated columnar epithelium consistent with CPAM. Arrows: rhabdomyomatous dysplasia. (**D**) Histological features Case 3, Desmin immunostaining, original magnification 10X. Irregularly shaped airways cystic spaces lined by pseudostratified ciliated columnar epithelium consistent with CPAM. Beown staining: rhabdomyomatous dysplasia.

Histopathological examination of the surgical specimens of all cases revealed hybrid lesions. Indeed, they all displayed adenomatoid cystic components within nonfunctioning lung tissue supplied by one or more aberrant vessel (Figure 3).

Classic hematoxylin-eosin staining was performed on all cases, identifying normal alveoli surrounded by inflammatory cells and cystic airspaces. Aberrant vascular walls were described concomitantly.

Case 3 specimen was also submitted to desmin immunostaining to search for rhabdomyomatous dysplasia, suspected due to the complete inclusion of the lesion within diaphragmatic fibers.

## 3. Discussion

CLMs are rare malformations, arising during the embryonic development. BPS are considered to be the second most common CLM, as they account for the 0.15%–6.40% of all CLM (7–9). They consist of non-functioning embryonic lung tissue, suopplied by one or more systemic arteries, thought to arise from an accessory tracheobronchial bud originating from the foregut (10). Among BPS, ILSs are the more frequent (75% of all BPS), they are located within the thorax, in the lower lung lobes, preferentially on the left side, while ELSs are rarer (25% of all BPS) and are mostly seen postero-medially, in the left lower chest (7). Ectopic ELS can be occasionally found within the abdominal cavity, most commonly in the left suprarenal area (11), and extraordinarily within the diaphragm. IDEPS account for 10%–15% of all ELS (12). The developmental mechanisms behind IDEPS are complex and still poorly understood. The lesion is thought to originate from the fusion of an accessory bud arising in close proximity to the diaphragm with embryological elements of the diaphragm.

The peculiarity and the exceptionality of this location carries great importance from a diagnostic and surgical point of view. Indeed, in many cases only surgical exploration can properly and correctly clarify the nature of the lesion, assuming not only a therapeutic role but also a diagnostic one. Differential diagnosis of an intradiaphragmatic mass involves a wide range of conditions and the anatomical relationships of this peculiar location hinder a straightforward verdict, requiring high expertise to orient within the broad spectrum of possibilities. Primary diaphragmatic tumors are an exceptionally rare finding, mostly being benign (13). The latter most commonly include lipomas, fibromas or bronchogenic cysts (14). The diaphragm may also be the site of invasion from adjacent malignancies such as mesothelioma or tumors of the liver (14). Given the rarity of the condition, poor literature exists regarding their diagnostic workup and surgery remains the mainstem of management.

The exceptionality of IDEPS makes it difficult to reach consensus regarding the best approach and technique for their management. Some authors advocate the thoracoscopic approach (3, 4, 15) because they were able to identify the lesion only once in the thorax, while others (16, 17) could identify the lesion only through the laparoscopic approach. Because of these controversy, some authors promote a combined thoracoscopic and laparoscopic approach (18, 19). Our series, although limited in numbers, supports the thoracic approach to identify and remove the lesion.

The first patient underwent thoracotomic IDEPS resection and left lower lobectomy, the second was started in thoracoscopy but required conversion to thoracotomy, in the third patient, resection and diaphragmatic reconstruction were eventually completed through thoracoscopy. In the first patient, after discussing with the parents the pros and cons of a lobectomy during the same thoracotomy to resect the IDEPS versus the risk of a second surgery should the emphysema had become symptomatic, the former approach was elected and pursued. It is possible that age at surgery may have had an influence on the possibility to avoid conversion. In fact, the second patient who required conversion was younger (and smaller) that the third that did not require conversion. In this last patient we started through a laparoscopic approach due to the preoperative imaging evidence of feeding vessels originating in the abdomen. In fact, we were able to identify and ligate part of the feeding vessels laparoscopically, However, the main feeding vessel and the lesion were detected only after conversion to thoracoscopy. This approach also allowed us to isolate it from the diaphragmatic muscle, to clip and resect safely the main feeding vessel and to repair the diaphragm. The only concern for the thoracoscopy only approach is that, when closed through the thorax, aberrant feeding vessels will rapidly retract within the abdominal cavity and bleeding may not be spotted immediately, should it occur. Having ports both in the chest and the abdomen may facilitate the hemostasis and management of bleeding, thereby supporting the combined approach. Also in our small series, the change from thoracotomy to thoracoscopy was associated with a reduction of post-operative length of stay, while remaining a safe and effective approach.

In all our patients, the histopathological analysis of resected IDEPS showed hybrid lesions with CPAM elements, with rhabdomyomatous dysplasia in one. As shown also by our series of CLM, CPAM elements are more frequently associated to ILS then ELS (10, 20–26). Hybrid lesions are defined carrying features of CPAM (both at histology and at imaging evaluation) within a portion of pulmonary tissue separate from the airways (BPS) and anomalous blood supply. The possibility of lesions characterized by histopathological features of both CPAM and BPS suggest a potential common etiopathogenetic mechanism between these two entities, and that the separation line between them is somewhat nuanced. As a consequence, the presence of hybrid lesions in all IDEPS is a peculiar finding which strengthens the indication for surgical removal of these lesions. BPS are rarely associated with malignancies and the exact underlying mechanism of malignant transformation is still unknown (27). Several hypotheses exist, but the etiology of the malignancies in BPS cases is believed to be multifactorial, including some effect of chronic inflammation. The presence of CPAM elements within the BPS may add to the risk of malignant transformation of these lesions (28–30). This may be particularly true in IDEPS bearing CPAM elements, when considering the findings from a recent study by Monteagudo et al. (31) who showed a correlation between CPAM proximity to the diaphragm and presence of rhabdomyomatous dysplasia, that is considered by some authors as potentially the first stage in the development of a pulmonary rhabdomyosarcoma (32, 33).

## 4. Conclusions

IDEPS are exceedingly rare CLM. The presence of CPAM elements within the lesions in all our patients, one of which with rhabdomyomatous dysplasia, supports their surgical removal. Our small series favors the thoracic approach, ideally thoracoscopy, as the optimal one, although the possibility to have a combined laparoscopic approach may allow a safer control of possible bleedings from main feeding vessels when they arise from the abdominal Aorta. Given the rarity of IDEPD, a larger, multicenter series may help to better characterize their nature and management.

## Data Availability

The raw data supporting the conclusions of this article will be made available by the authors, without undue reservation.
